# Professional quality life of psychiatry residents in Tunisia

**DOI:** 10.1192/j.eurpsy.2024.1663

**Published:** 2024-08-27

**Authors:** R. Masmoudi, S. Ajmi, A. Guermazi, F. Guermazi, I. Baati, J. Masmoudi

**Affiliations:** Psychiatry A, Hedi Chaker University Hospital, Sfax, Tunisia

## Abstract

**Introduction:**

The professional quality of life for psychiatry residents is a complex and multifaceted aspect of their careers. However, the demanding nature of their work can place significant stress on their own psychological well-being. Balancing the need to care for patients while also managing personal and professional responsibilities can be challenging. Nevertheless, psychiatry residents have the opportunity to make a profound impact on the lives of their patients and find fulfillment in their work.

**Objectives:**

To assess the prevalence of burnout (BO) and secondary traumatic stress (STS) among psychiatry residents.

**Methods:**

We conducted a descriptive online cross-sectional survey in January 2022 among psychiatry residents practicing at Hedi Chaker University Hospital in the Sfax region in Tunisia. Professional life quality was evaluated using The Professional Quality of Life Scale - 5 “ProQOL-5”.

**Results:**

The total number of residents was 34, of which 91.2% were female. Their mean age was 27.94 years±2.43. They were single in 67.6%. They were residents in adult psychiatry in 61.8% and in child psychiatry in 39.2%. For 91.2% of them, the specialty of adult or pediatric psychiatry was their own choice. The individuals had been practicing psychiatry for an average of two years. They reported a personal medical or surgical history, a personal psychiatric history, and a family history of psychiatric disorders in 32.4%, 8.8%, and 50%, respectively.

On the ProQOL-5 scale, we found that 88.2% of the residents had a moderate level of compassion satisfaction, 67.6% had a moderate level of burnout, and 52.9% had a moderate level of secondary traumatic stress.

**Conclusions:**

Our study showed a moderate professional life quality among psychiatry residents, hence the importance of implementing intervention strategies.

**Disclosure of Interest:**

None Declared

